# New technology and potential for telemedicine in battlefield brain injury diagnostics

**DOI:** 10.2217/cnc-2016-0014

**Published:** 2016-10-14

**Authors:** Leila H Eadie

**Affiliations:** 1 Centre for Rural Health, University of Aberdeen; Centre for Health Science, Old Perth Road, Inverness IV2 3JH, UK

**Keywords:** diagnosis, prehospital, remote support, telemedicine, ultrasound

Traumatic brain injury (TBI) is a considerable problem for the military worldwide, and the media have designated it the ‘signature injury’ of the conflicts in Afghanistan and Iraq [1]. It can occur following head trauma after a direct impact or from being in close proximity to a blast. TBI can have different forms depending on the extent of the injury; this editorial will focus on TBIs that result in brain hemorrhage. On the battlefield, it is likely to be overlooked in favor of more readily visible injuries, yet bleeding and inflammation within the head can cause death or pervasive ongoing psychiatric conditions.

## The problem

TBI affects mainly ground-based troops coming into contact with explosions, with more than 4% of those serving in the US military being diagnosed with TBI each year [2]. Most were seen in the army and Marine Corps, and approximately 77% of TBIs were ‘mild’. Similarly, a study looking at a sample of UK servicemen and women deployed in Afghanistan found a TBI rate of 2.1% in front line combat personnel and a 1-year incidence rate of 3.2% overall [3]. However, these figures are likely to be the tip of the iceberg as many service members are undoubtedly going undiagnosed with mild TBI that has the potential to lead to serious long-term health and socioeconomic consequences [4]. Ongoing symptoms include a range of physical, cognitive and emotional effects, often persisting long-term and resulting in unemployment, relationship difficulties and reduction of independence. The effects vary depending on the severity of injury, the brain area affected and whether it is a lone or repeat injury: there is evidence that multiple TBIs have a cumulative impact [5].

TBI's main insult to the brain is secondary to the primary injury following mechanical trauma. In severe cases, this secondary injury occurs due to bleeding and inflammation, having several different effects: contact with blood causes brain tissue to swell (cerebral edema), and pooled blood within the confines of the skull also puts pressure on nearby tissue, constricting blood flow and depriving the brain of oxygen, killing neurons and leading to a chemical cascade that reinforces the injury [6]. People suffering from TBI can deteriorate suddenly and die, and in some cases swift treatment can help reduce mortality. Others will have minor initial symptoms, yet untreated brain hemorrhage can have insidious long-term effects. The etiology of postconcussive syndrome is debated, but may be caused by diffuse axonal injury or persistent metabolic alterations resulting in neuronal dysfunction [2] and develops in 38–80% of patients with TBI [7].

While treatment for TBI involving hemorrhage will depend on the cause, location and amount of bleeding, it generally involves stopping the bleeding and relieving the pressure. Decompression can be achieved via surgery: craniotomy or burr-hole procedures allow the hemorrhage to be drained; or in less urgent cases, medications can be given to help reduce swelling and prevent seizures. Prompt diagnosis and early prehospital care can mean that additional injury can be prevented, if hemorrhages can be tapped blood, oxygen flow to the brain maintained and pressure controlled. Also of great importance is avoiding ‘second impact syndrome’ – a controversial condition in which additional TBI within weeks of a previous incident can result in diffuse cerebral swelling, brain herniation and potentially death [8] – by removing affected soldiers from active duty where they would be at risk of further TBI until the danger period has passed and the brain has had time to recover.

## Diagnosis

Key to the ability to successfully manage TBI is diagnosing the problem in the first place. Medics have to confirm that brain injury exists to be able to triage and offer treatment options. This is where battlefield clinicians are at a disadvantage: CT and MRI scanners are not often found in field hospitals, usually located a good distance away from a hospital with full facilities. So beyond observation of clinical symptoms, what options does a battle medic have to help diagnose a closed TBI as quickly as possible after the injury?

Questionnaire-based screening tools are available, such as the Military Acute Concussion Evaluation, comprising cognitive results, neurologic exam and symptoms [9], and the Warrior Administered Retrospective Casualty Assessment Tool [10], but these are generally administered long after the actual injury to confirm whether a concussion took place.

Diagnosis of any kind of head injury on the battlefield – or any prehospital situation – is not an easy matter. At the moment, the injured person can only be assessed using the observed clinical symptoms, which can be followed up with full scans when they arrive at a fully equipped hospital. Ambulances containing mobile head CT scanners have been constructed and trialed [11], but these are expensive, experimental and not currently practical, restricted in their range and requiring expert staff on board. Image quality is also not as good as that produced by hospital-based scanners.

## New options

There are other options being investigated, including infrared and microwave head scanners, Doppler and B-mode (grayscale) ultrasound.

The InfraScanner (InfraScan Inc., USA) is a handheld tool to screen for blood close to the skull: it can image to a depth of around 2.5 cm into the brain using near-infrared imaging to scan for hemoglobin, which is then compared with the opposite side of the head as baseline. It was developed with support from the US military and is now deployed in Marine Corps standard trauma kits. With sensitivity of 89.5% and specificity 81.2% this is obviously a useful device, despite not being likely to locate deeper situated bleeds [12].

A head scanner using Doppler ultrasound is in development: Neural Analytics (USA) is using machine learning techniques to analyze the morphology and pulsatility information from Doppler waveforms to detect concussion. Initial tests suggest this early stage system already has a sensitivity of 71% and a specificity of 83%, with research ongoing [13]. Another research-only head scanner using microwaves is currently undergoing testing in Sweden, with the aim of being used prehospital to diagnose stroke – differentiating between ischemic and hemorrhagic stroke [14].

But possibly the best bet could be ultrasound, well known as a useful emergency medicine tool and one deployed medics are likely to already have in their armamentarium; it is possible that this could be put to work diagnosing TBI. Transcranial ultrasound is performed through ‘acoustic windows’ where the skull is thinnest, and is not currently a commonly performed scan. It can be difficult to interpret and so expert help may be required, which is where communications systems and telemedicine can be useful.

Remotely supported imaging – where a nonexpert user performs scans guided by an expert, who also interprets the resulting images – can allow the use of technologies such as ultrasound, which would usually require substantial training and experience, in areas where it would not be cost effective to provide such expertise full time.

In addition to having an expert available as a guide, there are other ways to support novice ultrasound users, whether by keeping things simple and providing well defined, step-by-step imaging protocols or by using software support such as my own recent UK Ministry of Defence-sponsored project to help users track which parts of the brain they have imaged, and which are yet to be scanned. This program also creates a 3D image from the scans which can be transmitted to experts for interpretation [15].

Remotely supported point-of-care ultrasound has been trialed in extreme situations, such as in the Arctic Circle [16] and on the International Space Station [17]. It has been successfully used to guide children and people who have never performed any medical procedures before [18]. Remotely supported transcranial ultrasound has only recently been tested on healthy volunteers in a small study in the Scottish Highlands [19], where transport times to hospital can be large, and patients face many of the same problems as injured soldiers in terms of access to medical facilities. The tests, which involved transmitting live ambulance-based ultrasound to a hospital, proved the idea was feasible and a larger trial of remotely supported ultrasound is currently being initiated. We have started collecting images of patients with brain hemorrhage, intending to visualize various causes and locations of bleeds. Ultrasound also can be beneficial in further assessment of the head, able to diagnose skull fractures and assess intracranial pressure via elasticity measures, showing mass effect and midline shift, obliteration of the basal cisterns, and can provide evidence of herniation. It has potential to be a most useful tool, given support from expert sonographers.

## Conclusion

Medicine, and specifically diagnosis of TBI in areas remote or cut off from centers of care – such as the battlefield hospitals supporting the front line – is a difficult prospect. However, with newly developed technology and reassessment of older technologies such as ultrasound for new purposes, the battlefield medic can offer improved diagnostic abilities without requiring lengthy training. Ultrasound is already a useful tool in emergency medicine and also has the potential to assist with TBI diagnosis and triage where CT and MRI are not available. People already have visions using micromachined ultrasound chips to create a scanner that offers a portable ‘window’ into the body, also featuring automated diagnoses [20].

Back in the present day, modern communication technologies are such that streaming scans live to hospital-based experts and receiving guidance back can be readily achieved, meaning that any novice can record scans and receive immediate specialist support. In this way, there is hope that soldiers can be more promptly diagnosed and treated, and then removed from active duty in order to recover fully, reducing the risks associated with repeat TBI and other ongoing sequelae of the injury.
